# Odorous House Ants (*Tapinoma sessile*) as Back-Seat Drivers of Localized Ant Decline in Urban Habitats

**DOI:** 10.1371/journal.pone.0113878

**Published:** 2014-12-31

**Authors:** Adam Salyer, Gary W. Bennett, Grzegorz A. Buczkowski

**Affiliations:** Department of Entomology, Purdue University, West Lafayette, IN 47907, United States of America; Field Museum of Natural History, United States of America

## Abstract

Invasive species and habitat disturbance threaten biodiversity worldwide by modifying ecosystem performance and displacing native organisms. Similar homogenization impacts manifest locally when urbanization forces native species to relocate or reinvade perpetually altered habitat. This study investigated correlations between ant richness and abundance in response to urbanization and the nearby presence of invasive ant species, odorous house ants (*Tapinoma sessile*), within its native region. Surveying localized ant composition within natural, semi-natural, and urban habitat supported efforts to determine whether *T. sessile* appear to be primary (drivers) threats as instigators or secondary (passengers) threats as inheritors of indigenous ant decline. Sampling 180 sites, evenly split between all habitats with and without *T. sessile* present, yielded 45 total species. Although urbanization and *T. sessile* presence factors were significantly linked to ant decline, their interaction correlated to the greatest reduction of total ant richness (74%) and abundance (81%). Total richness appeared to decrease from 27 species to 18 when natural habitat is urbanized and from 18 species to 7 with *T. sessile* present in urban plots. Odorous house ant presence minimally influenced ant communities within natural and semi-natural habitat, highlighting the importance of habitat alteration and *T. sessile* presence interactions. Results suggest urbanization releases *T. sessile* from unknown constraints by decreasing ant richness and competition. Within urban environment, *T. sessile* are pre-adapted to quickly exploit new resources and grow to supercolony strength wherein *T. sessile* drive adjacent biodiversity loss. Odorous house ants act as passengers and drivers of ecological change throughout different phases of urban ‘invasion’. This progression through surviving habitat alteration, exploiting new resources, thriving, and further reducing interspecific competition supports a “back-seat driver” role and affects pest management strategies. As demonstrated by *T. sessile*, this article concludes native species can become back-seat drivers of biodiversity loss and potentially thrive as “metro-invasive” species.

## Introduction

Habitat alteration and invasive species are commonly cited as the main causes of biodiversity loss [1], [2]. Estimating the relative importance of these factors is essential when making appropriate conservation decisions and directing resources towards eliminating the primary cause of species extinctions. Habitat alteration is thought to be the primary factor affecting biotic homogenization and localized species extinctions, and species invasions are thought to have a lesser, secondary role [1]–[3]. These two factors often overlap making it difficult to determine their relative contribution, if any, to localized extinctions. Several studies question the importance of species invasions, and propose invasive species are not drivers of biodiversity loss, but rather are passengers of more fundamental change in the ecosystem [4]–[6]. Within ants (Fomicidae), King and Tschinkel [7] propose that invading red imported fire ants, *Solenopsis invicta*, fit the passenger model as secondary reducers of biodiversity. Although fire ant presence reduced native ant abundance by 90% and richness by 70% [8], these impacts were found only in habitats with prior anthropogenic disturbance [7]. In contrast, Argentine ants, *Linepithema humile* have invaded diverse, undisturbed habitats as primary drivers of ecological change [9]. By and large, studies suggest human activity is the primary factor responsible for species invasions [7], [10]–[12]. Additional research needs to de-couple invasion and habitat alteration by examining indigenous species intolerant of ecological change.

Ants are a diverse group of species that can positively respond to urbanization through growth or expansion [13]–[15] and negatively through loss in biodiversity [16]–[18]. Subtle levels of habitat disturbance can manifest via compositional changes in ant communities [14], making ant diversity an ideal model for gauging the impact of anthropogenic disturbance on local biodiversity [19]. As natural habitats become urbanized, ant diversity declines and many ants that perform ecosystem services are lost [14]. The loss of these cryptic ants and their functional roles can generate indirect ecological consequences such as disrupting local ecosystems [20]–[21] and surrendering formerly exploited resources. Results from a recent study contradicted this well-supported association between urbanization and ant homogenization [22], suggesting unknown factors contribute to ant survival in urban habitat.

While ant species diversity typically declines from natural into urban habitats [23]–[25], certain species tolerate urbanization and benefit from it by monopolizing remaining resources with minimal competition [14]–[15], [26]. Urban disturbance specialists are categorized as opportunists, whereby these species share flexible nesting and feeding habits, pre-adapting them to a variety of urban environments [27]. Upon successful urban invasion, ants can quickly grow into pests by monopolizing resources [28]–[30] or by exhibiting tramp behaviors such as polydomy, extreme polygyny, reduced conspecific aggression, and colony spread by budding [31]. Numerous studies have measured the individual importance of these behaviors to better understand invasive species spread [32]–[35]. However, none have tracked an invasive species from its human commensal start as an urban specialist. Continued research is needed to identify what factors enable invasive species' dominance and to determine the comparative harm generated by invasion and habitat disturbance against biodiversity conservation efforts.

This study investigated driver and passenger models within odorous house ants (*Tapinoma sessile*), a native ant that flexibly expresses a variety of invasive behaviors [15], [26]. The relative impact of habitat alteration and *T. sessile* presence on native, neighboring ant nests was correlated by sampling a variety of habitats in the presence and absence of *T. sessile.* Urban and natural dwelling odorous house ants are the most widespread, ecologically tolerant ants native to North America [36]–[37] and a newly invasive species in Hawaii [38]. Odorous house ants share generalist characteristics and adaptably express many structures and tramp species behaviors [15], [26]. In natural habitats, *T. sessile* colonies are typically small, monogyne, monodomous, and subdominant [26]. Urban colonies of *T. sessile* are attributed with polygyny, polydomy, and dominance with supercolony sizes reaching millions [15], [26]. Odorous house ants that survive urbanization of their native natural habitat often proceed to burgeon into ecologically dominant pest species [39], potentially experiencing a passenger benefit from habitat disturbance. Despite diminished urban ant diversity relative to natural habitats, preliminary evidence suggests that odorous house ants further reduce ant diversity in urban habitats [26]. Therefore, *T. sessile* is an ideal species to investigate localized biodiversity loss through driver or passenger models. The mechanism behind *T. sessile*'s successful displacement of other ants is unknown and could contribute to understanding urban ecology, pest management, and invasive species ecology.

## Materials and Methods

No permits were required for fieldwork performed in this study. Surveys took place on public and residential properties. All residential property owners approved of surveying methods prior to this study. No protected or endangered species were collected or harmed to complete this study.

### Study sites and research plots

This study was conducted within a 15 kilometer radius of Purdue University in West Lafayette, Indiana, U.S.A. To estimate the effect of habitat alteration (urbanization) on biodiversity loss, ant species diversity and abundance were determined in three distinct habitats: natural, semi-natural, and urban. Natural habitats consisted of forests larger than 2 continuous hectares with little to no anthropogenic disturbance, and dominated by mature trees. Semi-natural habitats were natural habitats that showed moderate signs of anthropogenic influence and included parks, fields, and forest edges. Urban habitats were highly urbanized commercial or residential locations. To estimate any ‘driver’ ant influence, plots with *T. sessile* present or absent were sampled in each habitat type. Sixty plots were sampled in each habitat: 30 with *T. sessile* neighboring colonies present and 30 with *T. sessile* absent. In total, 180 plots were surveyed across all habitats: 90 with *T. sessile* present and 90 with *T. sessile* absent. Plots with *T. sessile* present were located by randomly searching each habitat for visual signs of *T. sessile* trailing activity, inspecting debris on the ground, or following workers from baits (jelly) back to their nests. The nest location then served as the focal point for a 2.75 by 2.75 m sampling plot. Any additional *T. sessile* nests discovered in the preliminary plot were tested for signs of aggression against the original *T. sessile* focal colony. If no aggression was seen after five 1-minute replications of introducing opposing workers into an inner Fluon^TM^-coated 27 mm diameter by 60 mm glass vial, the nests were considered part of the same colony. Plots with T. sessile absent were haphazardly assigned within 15–40 m of plots with T. sessile present. Care was taken to assure that paired habitat plots were similar in habitat features such as levels of development, plant cover, and insolation. Ant species diversity and abundance were determined in all plots by exhaustively sampling approximately 45 minutes within each plot for ants. Meticulous hand-sampling was utilized for this study because pit-fall trap surveying can yield variable results for ant collecting [40]–[42]. All above- and below-ground debris was carefully inspected to account for hypogaeic and epigaeic ants and all ants were identified to species using published keys [43]–[46]. Hand-sampling included overturning all rocks and splitting, opening, and bumping of any possible nesting objects found within the site (branches, logs, nuts, cans, pens, debris) to search for inconspicuous nests and encourage the evacuation of ants. A garden hoe was used to scrape off tree bark for any suspected nests and to dig up a minimum of 3 inches of soil throughout the entire plot in the search for ants. Nests of arboreal ant species were considered present when a steady trail of ants could be observed on a tree trunk. Sampling was conducted during the day (8:00AM–5:00PM) between May-August during 2009 and 2010. Although total sampling occurred over 2 summers, each forest, park, or urban region was sampled during one summer to prevent resampling a newly redistributed plot of ants. Within each plot ant abundance was determined by counting the total number of nests present in the plot and ant diversity was determined by counting the total number of species in the plot. Although possible, it is unlikely T. sessile ants from present plots foraged as far as the haphazardly assigned absent plots. This study focused on the immediate impacts to neighboring ant colonies localized near the T. sessile colony. For the purpose of comparing ant species diversity in plots with T. sessile presence or absence, odorous house ant counts were not included in plot tallies when computing the analysis.

### Statistical Analysis

ANOVA testing (SAS, V9.2, Copyright SAS Institute Inc. 2008) (PROC GLIMMIX) was used to test for differences in nest abundance or species diversity between habitats and accounted for random effect of paired sites. A Poisson regression model was used to calculate the significance of effects including habitat type, odorous house ant's numerical and categorical presence, and interaction effects on native ant nest and species counts. Comparisons made of average species or nest counts within treatments of either *T. sessile* present or absent were analyzed using multiple comparisons, Tukey-Kramer adjustment, with a family-wise significance of 0.05. Pair-wise comparisons of total nest and species counts within study and control sites were calculated through PROC GLIMMIX Restricted Maximum Likelihood analysis. Simpson index, ant species diversity (Shannon index), and ant species equitability were assessed for each combination of factors.

## Results

**Figure 1:**
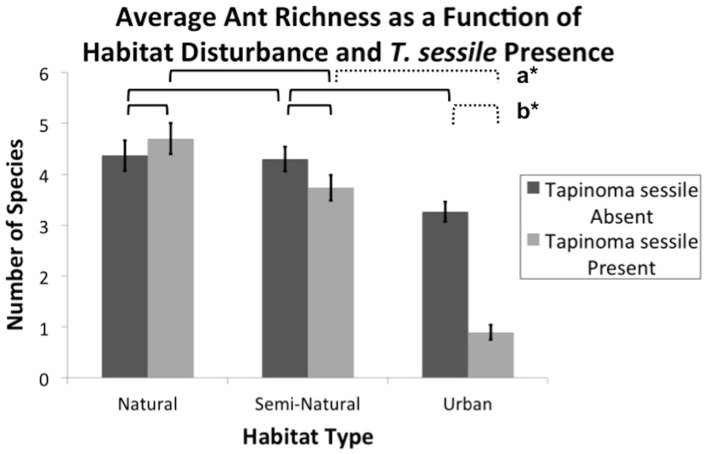
Relative impact of habitat alteration and *Tapinoma sessile* presence on average ant richness. Bars at the column peaks indicate standard error. Brackets indicate an analysis of average species differences between columns under the bracket ends. Solid brackets denote non-significant difference of species average. Dotted brackets followed by a bolded letter and asterisk denote significantly different species averages. Differences between average species counts were calculated using multiple comparisons, Tukey-Kramer adjustment. a*: SE = 0.21; df = 87; Adj. P<0.0001. b*: SE = 0.22; df = 87; Adj. P<0.0001.

A total of 45 ant species representing 894 nests were discovered in 180 plots (Table 1). These species comprised of 20 genera in 4 subfamilies. Both *T. sessile* presence and urbanization negatively correlated with adjacent ant community richness and diversity. Habitat type (F_2,87_ = 31.85, P<0.0001), *T. sessile* presence (F_1,87_ = 24.55, P<0.0001), and their interaction (F_2,87_ = 14.87, P<0.0001) correlated with significant changes in average ant richness (Fig. 1). No significant difference of average species richness was found between natural and semi-natural plots with *T. sessile* (SE = 0.13; df = 87; Adj P = 0.459) or without *T. sessile* (SE = 0.12; df = 87; Adj. P = 1.000). Natural plots with *T. sessile* neighboring colonies present (SE = 0.21; df = 87; Adj. P<0.0001), but not absent (SE = 0.13; df = 87; Adj. P = 0.261), correlated with increased species richness over urban habitat. Similarly, urban habitat correlated to a decreased richness compared to semi-natural habitat with *T. sessile* present (SE = 0.21; df = 87; Adj. P<0.0001), but not absent (SE = 0.13; df = 87; Adj. P = 0.261). Native ants experienced a significant decrease in diversity in plots correlated with neighboring *T. sessile* present (F_1,87_ = 24.55 P<0.0001). Within these plots, there was a significant difference in ant richness between all three habitat types (F_2,87_ = 31.00, P<0.0001), as well as between types with *T. sessile* absent (F_2,87_ = 3.18, P = 0.047). Upon further investigation, *T. sessile* presence did not impact richness within natural (F_1,87_ = 0.23, P = 0.630) or semi-natural (F_1,87_ = 1.48, P = 0.227) habitats but urban habitat experienced highly disproportionate differences in ant richness between plots with and without *T. sessile* (F_1,87_ = 35.18, P<0.0001). With *T. sessile* present, natural (F_1,174_ = 118.12, P<0.0001) and semi-natural (F_1,174_ = 65.67, P<0.0001) habitats revealed significantly more ant richness than found in urban plots. These findings were reflected when comparing natural (F_1,174_ = 11.13, P = 0.001) and semi-natural (F_1,174_ = 9.90, P = 0.002) plots to urban plots in absence of *T. sessile*. Urbanization of natural or semi-natural habitat plots with *T. sessile* absent correlated in the loss of 9 and 11 species respectively (Table 2). Additionally, urban habitat lost 11 species with *T. sessile* present.

**Table 1:** Ant species' numerical presence in response to treatments.

Name	Subfamily	Genus	N-	SN-	U-	N+	SN+	U+
*Acanthomyops claviger*	Formicinae	*Acanthomyops*	3	2		4	4	
*Aphaenogaster fulva*	Myrmicinae	*Aphaenogaster*	20	14		12	10	
*Aphaenogaster picea*	Myrmicinae	*Aphaenogaster*	5	7		2		
*Aphaenogaster rudis*	Myrmicinae	*Aphaenogaster*	12	13	3	31	15	
*Aphaenogaster tennennsis*	Myrmicinae	*Aphaenogaster*	4		1			
*Brachymyrmex depilis*	Formicinae	*Brachymyrmex*	1	3		1		
*Camponotus americanus*	Formicinae	*Camponotus*	2	1				
*Camponotus caryae*	Formicinae	*Camponotus*	4	3		3	6	
*Camponotus castaneus*	Formicinae	*Camponotus*			2	2	1	
*Camponotus chromaiodes*	Formicinae	*Camponotus*	4		5			
*Camponotus nearcticus*	Formicinae	*Camponotus*		2			2	
*Camponotus pennsylvanicus*	Formicinae	*Camponotus*	21	11	8	12	14	
*Camponotus subbarbatus*	Formicinae	*Camponotus*	10	8	2	6	2	
*Crematogaster cerasi*	Myrmicinae	*Crematogaster*	12	14	31	7	8	4
*Forelius pruinosus*	Dolichoderinae	*Forelius*			1		2	1
*Formica argentea*	Formicinae	*Formica*					2	
*Formica montana*	Formicinae	*Formica*		2			2	
*Formica neogagates*	Formicinae	*Formica*	1	2	1	3	2	
*Formica nitidiventris*	Formicinae	*Formica*	1	4				
*Formica pallidefulva*	Formicinae	*Formica*			1		2	1
*Formica subsericea*	Formicinae	*Formica*	10	4		9	3	
*Formica vinculans*	Formicinae	*Formica*			4			1
*Lasius alienus*	Formicinae	*Lasius*	1	4	1	6	5	
*Lasius claviger*	Formicinae	*Lasius*	8	3		2	3	
*Lasius neoniger*	Formicinae	*Lasius*	12	28	51	9	13	8
*Monomorium minimum*	Myrmicinae	*Monomorium*			2			
*Myrmica americana*	Myrmicinae	*Myrmica*		2			4	
*Myrmica fracticornis*	Myrmicinae	*Myrmica*	4	2		2	5	
*Myrmica latifrons*	Myrmicinae	*Myrmica*	1	1			1	
*Myrmica pinetorum*	Myrmicinae	*Myrmica*	13		6	4		1
*Myrmica spatulata*	Myrmicinae	*Myrmica*	6	4		9		
*Paratrechina parvula*	Formicinae	*Paratrechina*					1	
*Pheidole bicarinata*	Myrmicinae	*Pheidole*					2	
*Ponera pennsylvanica*	Ponerinae	*Ponera*	9	2	1	8		
*Prenolepis imparis*	Formicinae	*Prenolepis*		2	1	4	5	
*Protomognathus americanus*	Myrmicinae	*Protomognathus*				1		
*Solenopsis molesta*	Myrmicinae	*Solenopsis*		1	8		1	4
*Stenamma brevicorne*	Myrmicinae	*Stenamma*	1	2		1		
*Stenamma meridionale*	Myrmicinae	*Stenamma*	3	4		1		
*Tapinoma sessile*	Dolichoderinae	*Tapinoma*				37	34	69
*Temnothorax curvispinosus*	Myrmicinae	*Temnothorax*	19	12		33	14	
*Temnothorax duloticus*	Myrmicinae	*Temnothorax*	1	1		1		
*Temnothorax longispinosus*	Myrmicinae	*Temnothorax*	1					
*Temnothorax pergardei*	Myrmicinae	*Temnothorax*					1	
*Temnothorax texanus*	Myrmicinae	*Temnothorax*					1	
*Tetramorium caespitum*	Myrmicinae	*Tetramorium*		6	54		20	17

Numbers following species correspond to the number of nests found within the respective factor combinations. Absence of a number indicates absence of the respective species within factor combination. Column abbreviations N, SN, U correspond with natural, semi-natural, and urban habitats. Negative or positive column abbreviations indicate the absence (-) or presence (+) of *T. sessile*.

**Table 2:** Ant community composition in three habitats with and without *Tapinoma sessile*.

Habitat	*T. sessile*	Σ(R)^1^	Σ(A)^2^	R^3^	A^4^	D^5^	H'^6^	J'^7^
Natural	Absent	27 a, a	189 a, a	4.37 (0.30) a, a	6.30 (0.53) a, a	0.06	−1.15	−0.80
Semi-Natural	Absent	29 a, a	164 a, a	4.30 (0.24) a, a	5.47 (0.38) a, a	0.07	−1.27	−0.87
Urban	Absent	18 a, a	178 a, a	3.27 (0.19) a, a	5.93 (0.52) a, a	0.21	−0.84	−0.67
Natural	Present	25 a, a	178 a, a	4.70 (0.30) a, a	5.93 (0.45) a, a	0.09	−1.17	−0.83
Semi-Natural	Present	27 a, a	149 a, a	3.73 (0.25) a, a	4.97 (0.33) a, a	0.07	−1.23	−0.86
Urban	Present	7 b, b	36 b, b	0.90 (0.15) b, b	1.20 (0.19) b, b	0.28	−0.64	−0.76

1 Σ(R) is the total number of species found throughout all 30 sites within the treatment.

2 Σ(A) is the number of total nests found throughout all 30 sites within the treatment.

3 R is the average number followed in parentheses by the standard error of species found throughout the treatment.

4 A is the average number followed in parentheses by the standard error of nests found throughout the treatment. Values within columns followed by the same first letter are not significantly different from values found within the same treatment of *T. sessile* but among varying habitats. Values within columns followed by the same second letter are not significantly different from values found within the same treatment of habitat but among differing *T. sessile* presence.

5 *D* Simpson index,

6 *H′* ant species diversity (Shannon index), and

7 *J′* ant species equitability.

**Figure 2:**
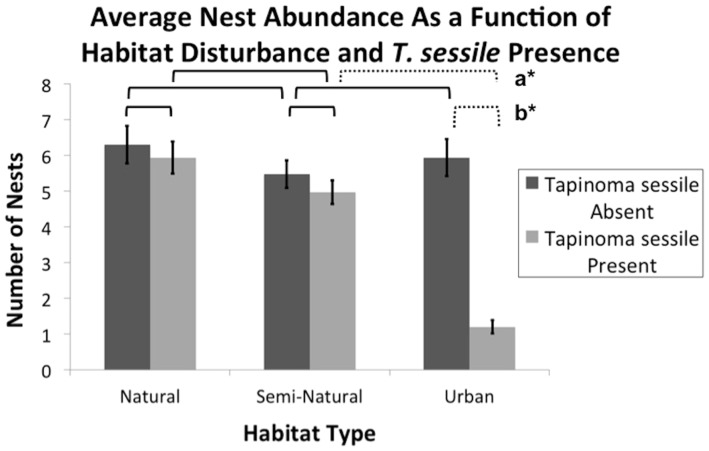
Relative impacts of habitat alteration and *Tapinoma sessile* presence on average ant abundance. Bars at the column peaks indicate standard error. Brackets indicate an analysis of average nest abundance differences between columns under the bracket ends. Solid brackets denote non-significant difference of nest abundance. Dotted brackets followed by a bolded letter and asterisk denote significantly different nest abundance averages. Differences between average nest abundance were calculated using ANOVA testing under PROC GLIMMIX. a*: SE = 0.21; df = 87; Adj. P<0.0001. b*: SE = 0.22; df = 87; Adj. P<0.0001.

The analysis of nest abundance reflected results similar to richness findings. Habitat type (F_2,87_ = 26.13, P<0.0001), *T. sessile* presence (F_1,87_ = 55.75, P<0.0001), and their interaction (F_2,87_ = 28.98, P<0.0001) were all significant when modeling average nest abundance. However, nest abundance differences only manifested when comparing across habitats in plots with *T. sessile* present (F_2,87_ = 36.07, P<0.0001) (Fig. 2). Urban habitat correlated significant differences in nest abundance between plots with and without *T. sessile* (F_1,87_ = 76.49, P<0.0001). Interestingly, high numbers of *T. sessile* were positively correlated with average interspecific nest abundance within urban habitat when compared to plots with few *T. sessile* nests (SE = 0.1049; df = 84; P = 0.049). Odorous house ant presence correlated to a diminished total interspecific nest abundance only under urban settings (F_1,174_ = 64.82, P<0.0001). Natural (F_1,174_ = 0.63, P = 0.428) and semi-natural (F_1,174_ = 0.93, P = 0.0.337) abundance were unaffected by neighboring *T. sessile*. With *T. sessile* present, natural (F_1,174_ = 64.82, P<0.0001) and semi-natural (F_1,174_ = 41.05, P<0.0001) habitats correlated significant decreases in total abundance when converted to urban habitat. A total of 178 nests within urban plots without odorous house ants decreased to 36 nests with neighboring *T. sessile*, correlating a significant decrease (F_1,174_ = 45.82, P<0.0001) of approximately 80% total abundance.

## Discussion

Odorous house ants and urbanization correlated with decreased ant richness and abundance but neither exclusive models (passenger or driver) appear to fit the results. Urbanization correlated with a diminished total ant richness from 27 to 18 species (−33%), while the presence of *T. sessile* further lowered ant richness from 18 to 7 species (−41%) in the immediate vicinity of *T. sessile* nests. These results contrast with Menke's [22] conclusions and further support richness decline caused by urbanization. Both *T. sessile* and urbanization interacted, correlating to a total ant richness loss of 20 species (−74%). The slightly larger reduction of richness correlated by *T. sessile* may suggest the effect of invasion is the primary driver of biodiversity loss while habitat alteration is secondary. Additionally, urban colonies of *T. sessile* correlated to a decrease in nearby average ant abundance from 5.93 to 1.20 nests per plot and total nest abundance from 178 to 36, which represents an 80% reduction of average and total nest abundance. Despite what appears to be a dominant, driver role in urban habitats, *T. sessile* presence does not impact ant richness or abundance within natural or semi-natural habitats. The absence of *T. sessile* impact within natural settings is likely due to its subdominant, minor role within native ant communities [47]–[48]. Previous research shows that high biodiversity is a barrier against invasions [49] and this theory could explain why *T. sessile* does not flourish into supercolonies within natural habitats. The odorous house ant is in the minority of species capable of tolerating urbanization [26], [50], and likely upon release from competitive species or natural habitat constraints capitalizes on vacant niches and becomes established as a dominant urban pest [26], [51] and an invasive species [38].

Driver and passenger labels fit *T. sessile* at different stages of colony development into supercolonies but neither model explains the overwhelming interactive effect of urbanization and *T. sessile* presence. Conversion of the natural environment into an urban landscape displaces native species, and creates a vacuum where vacant niches are slowly filled [52]. The adaptation of native species populating urban areas [51] and their shared traits has only recently received research attention [53]. Odorous house ants reap passenger benefits provided by habitat alteration when first invading urban landscapes as relatively small colonies. Over an unknown period of time, *T. sessile* colonies grow into supercolonies and likely exert a localized driver influence resulting in highly reduced species richness and abundance. Upon surpassing a population threshold, invasive species gain monopolization over resources, resulting in dramatic population expansion through positive feedback [29]. Odorous house ants likely surpass a similar population threshold when favorable conditions allow them to express invasive syndrome characteristics [15], [26], [51] rarely seen in natural habitats. Facultative expression of polygyny [26] appears to be a key attribute aiding urban *T. sessile*'s driver dominance. Advantages of polygyny can include faster spread or dispersal [54] and superior exploitation or interference of resources [55]. It remains unclear what triggers the expression of polygyny seen primarily within urban colonies of *T. sessile* [26]. Knowledge of when or why *T. sessile* switch to polygyny, polydomy, with increased interspecific aggression will strengthen any assessment of *T. sessile*'s threat to global biodiversity and how it fits within invasive models.

**Figure 3:**
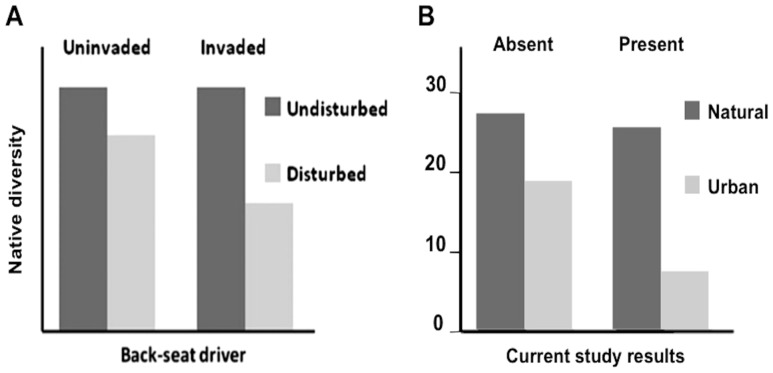
Comparison of archetypal back-seat driver data to *Tapinoma sessile* and urbanization influence on ant community richness. Both bar graphs represent studies sampling species richness within undisturbed or disturbed habitat in the presence or absence of an invasive species. (**a**) This chart is copied directly from Berman and Andersen [58] (Fig. 1) using ‘natural experiments’ to visualize data typical of a back-seat driver model. (**b**) The second chart represents total richness data plotted from this study and mirrors the idealized back-seat driver data. Numbers left of the Y-axis represent total species number.

Initial attempts to classify *T. sessile* as drivers or passengers failed because previous studies compared the exclusive and additive impacts of invasion and habitat degradation [6]–[7], [9]. However, a new invasive model described as “back-seat drivers” [56] fits *T. sessile*'s progression through colonization of urban areas, exploitation of urban resources, colony growth, followed by drastic reduction of ant richness. The back-seat driver model highlights the interactive effect of habitat disruption and invasive spread as observed in our study (Fig. 3) and seen in other invasive ants [57]. Ecosystem restoration can be achieved by resolving a single harmful factor under passenger and driver models, but requires controlling both disturbance and invader factors under a back-seat driver model [56]. Habitat disturbance through urbanization is an inherently permanent process that cannot be reverted for the purpose of pest species management. Therefore, management strategies should not be aimed at eliminating, but instead continuously controlling pockets of urban populations.

Although this study cannot directly assign a causality relationship between *T. sessile* and native ant decline, strong correlations provide support for such claims. An argument could be made that in urban areas *T. sessile* supercolonies do not drive out interspecific ants, but instead colonize areas where other urban ants are not found, thus negating the suggested back-seat driver influence. However, *T. sessile* colonies within this project were found nesting in areas with resources nearby, frequently available in abundance beyond possible exhaustion by odorous house ants. Research indicates urban ants may not be nest limited [57], but a large variety of vacant nesting sites paired with proximate, reliable, and frequently recurring food resources is likely to attract nearby colonization. These ideal locations are likely competed for leading to the dominant ant species monopolizing the territory. Urban territories adjacent to *T. sessile* nests supported fewer species and could be indicative of *T. sessile* back-seat driver influence within urban ant populations. If urban odorous house ant colonies continue to expand spatially or saturate the urban landscape, a plausible consequence would be the exclusion of interspecific competitors throughout urban habitat, impacting ants on a population level. Additionally, the relationship between increased numerical *T. sessile* presence and interspecific nest abundance within urban habitat would appear counterintuitive. This result is likely due to selective tolerance of a few urban species by odorous house ant colonies that have not reached supercolony dominance.

In conclusion, *T. sessile* can become back-seat drivers of ecological change under varying invasion contexts. As demonstrated with *T. sessile*, back-seat driver species receive a passenger's benefit from habitat alteration during early phases of urban colonization and are capable of localized displacement of indigenous species as a driver in the later stages of expansion. Under back-seat driver models, knowledge of the invasion phase will facilitate appropriate management practices, such as preventing potential intruders, eliminating initial propagules, and managing disturbances caused by established invaders. Early management practices, including reduction of food or housing resources, should prevent new invaders from reaching back-seat driver influence. Management practices following established invasion will vary given habitat type. Managing natural ecosystems will require expending resources on treating the invasive species and restoring the habitat to its original composition of fauna and flora. As described in this study, urban habitat management will involve continual treatment of invasive or pest species. Perhaps current invasive species began as pests saturating their respective urban environments. Native, urban-dwelling species are more likely to benefit from human-mediated dispersal due to trade and therefore could flourish as “metro-invasive” species. Future research should investigate any link between urban pest species and global invasive species with the goal of identifying and preventing metro-invasive spread.
